# Toward a More Relational Model of Sexual Minority Identity Concealment

**DOI:** 10.1007/s10508-022-02491-5

**Published:** 2022-11-28

**Authors:** David Matthew Doyle, Manuela Barreto

**Affiliations:** Department of Psychology, Washington Singer Laboratories, University of Exeter, Exeter, EX4 4QG UK

“Furthermore, the deadly elasticity of heterosexist presumption means that, like Wendy in *Peter Pan*, people find new walls springing up around them even as they drowse: every encounter with a new classful of students, to say nothing of a new boss, social worker, loan officer, landlord, doctor, erects new closets whose fraught and characteristic laws of optics and physics exact from at least gay people new surveys, new calculations, new draughts and requisitions of secrecy or disclosure.”

Eve Kosofsky Sedgwick, *Epistemology of the Closet* (1991, p. 68)

Across recent decades, scholars have devoted some effort to elucidating the psychological consequences of “the closet”—often defined as a period of total concealment of sexual minority identity. Both popular representations (e.g., films such as *Love, Simon*, and *Brokeback Mountain*) as well as academic conceptual models (e.g., Jackson & Mohr, 2016; Rosario et al., 2001) have portrayed the uniquely stressful and formative experiences that might characterize such periods of life. In an admirable extension of this work, in their Target Article, Pachankis and Jackson (2022) propose a developmental model of the closet that outlines specific stressors and psychological adaptations that occur during various periods of the closet, defined as the “pre-closet,” “closet,” and “post-closet.” While this novel model attempts to incorporate social structures and contextual factors, the developmental model of the closet is ultimately highly focused on individual experience and psychology (i.e., intrapersonal processes), often agnostic to the relational nature of these experiences and their enduring sequelae. We have recently called upon researchers interested in social stigma—including sexual minority stigma—to more explicitly acknowledge and attend to the effects of stigma on social relationships and to the potential contributions of relationship science to this field (Doyle & Barreto, in press). Beginning from the premise that a more relational model of sexual minority identity concealment is needed in order to advance the field, in the sections that follow we discuss the relational nature of secrets and implications for conceptualizing concealment and disclosure for sexual minorities—highlighting the importance of the process of identity management—as well as the relational (and contextual) nature of identities and how self-presentation goals and social feedback shape authenticity for sexual minorities.

## Relational Nature of Secrets and Identity Management

The nature of secrets, along with concealment and disclosure, necessitates a consideration of social relationships. Secrecy is inherently a relational phenomenon (Bedrov et al., 2021)—one cannot hold a secret without another person or persons to whom one is unwilling or unable to acknowledge something, irrespective of whether that secret is actually already known by others or not. In the case of sexual minorities, the closet may be created through an unwillingness or inability to acknowledge to others one’s sexual attractions, behaviors, fantasies or identity (Salomaa & Matsick, 2019) for various reasons, including but not limited to fear of rejection, harassment and discrimination or a wish to maintain privacy regarding one’s romantic or sexual life (Schrimshaw et al., 2018). It is worthwhile noting that the importance of these various motivations may shift from one situation to another (e.g., greater fear of discrimination at work as compared to at home) as well as within a person over time (e.g., greater desire for privacy in adulthood as compared to in youth).

As the quote by Sedgwick from her seminal theorizing on the closet highlights, rather than there being a simple binary of *in* versus *out* of the closet, each new social encounter necessitates decision-making regarding disclosure and concealment. As such, we (e.g., Doyle, 2022; Doyle & Molix, 2016; Newheiser & Barreto, 2014), along with others (e.g., Jones & King, 2014; E. B. King et al., 2017; Schmitz & Tyler, 2018), have pushed for coming out to be understood not as a discrete event but as a process of identity management in members of stigmatized groups. The notion of identity management acknowledges that members of stigmatized groups are active agents who negotiate self-presentation strategies by considering features of their social environments and the broader contexts in which they are embedded. In addition to concealment and disclosure, other identity management strategies that relate to the closet include covering (i.e., downplaying rather than hiding one’s identity in order to keep it from “looming large” in interactions; Doyle & Molix, 2016; Yoshino, 2006) and tacit acknowledgement of a stigmatized identity (i.e., an “open secret”; Sedgwick, 1991; Villicana et al., 2016). From this perspective, one would struggle to speak about a single act of coming out, but one would also not necessarily speak in terms of the “outness gradient” that Pachankis and Jackson (2022) argue against (i.e., a continuum from lesser to greater outness). Instead, one would speak of repeated identity negotiation that plays out in each new context where the identity is salient—potentially creating what has been termed “disclosure disconnects” (Ragins, 2008).

Importantly, while Pachankis and Jackson (2022) assume that there is something unique and formative about the first time one discloses, the first disclosure is not necessarily the most meaningful or impactful. Instead, research has indicated that a range of factors predict the outcomes of disclosure—other than whether or not it is the first one—such as the reaction of the person or people to whom one discloses (Chaudoir & Fisher, 2010; Griffith & Hebl, 2002; Law et al., 2011). Indeed, often the first disclosure is made to someone, and in an environment, that is (expected to be) safe and supportive (e.g., Chaudoir & Quinn, 2010; W. S. Ryan et al., 2015) and therefore its impact might be quite different from later disclosures to people who might be less accepting, or in environments that might be more intimidating.

The developmental model of the closet focuses heavily on structural and contextual factors that shape concealment, which is welcome since these kinds of factors are often ignored in research. However, features of specific dyadic or small group (e.g., family) relationships are often central to disclosure decisions as well as to responses to disclosure and thus also to well-being after disclosure. That is, not all individuals living under the same structural conditions, whether at the country-, region- or neighborhood-level, respond the same way to disclosure. Responses to coming out are as much a product of the particular nuances of family functioning as they are of the social structure in which the family is embedded (Baiocco et al., 2015; Willoughby et al., 2006, 2008). For example, some gay men who hesitated to come out to their mothers report that they did so because they did not want to add stress to the mothers’ already difficult lives (Valentine et al., 2003). Sexual minorities may live in countries or cities with relatively low levels of structural stigma, but rejection by family, often as a result of pre-existing family instability and conflict, can still lead to extreme negative consequences, including homelessness (e.g., Castellanos, 2016; McCann & Brown, 2019) and suicidality (e.g., Ryan et al., 2009; VanBergen & Love, 2022). Similarly, even in societies that are widely accepting of homosexuality, there are families that are less accepting, often because of specific cultural or religious beliefs or experiences, which do not always match those of the wider social structure. Crucially, research demonstrates that motivations and needs of both disclosers as well as confidantes shape outcomes after disclosure (Foster & Talley, in press). For example, coming out in relationships marked by autonomy support rather than control is consistently associated with better well-being after disclosure (Legate et al., 2012; Ryan et al., 2017).

Beyond considering the relational nature of secrets and identity management, we have also previously argued that it is critical to evaluate how stigma influences social relationships; that is, considering social relationships as an outcome of stigma-related processes, such as identity concealment or disclosure (Doyle & Barreto, in press). How a person reacts to disclosure doesn’t just pattern the discloser’s own psychological adaptations, it also affects those who are disclosed to and shapes the relationship itself, for better or for worse (Clair et al., 2005; Phillips et al., 2009; Valentine et al., 2003). Consequently, close relationship functioning has powerful bidirectional associations with mental health and well-being (Doyle & Link, 2022), part of which is linked to how relational partners respond to the disclosure of stigmatized identities. So, rather than viewing reactions to disclosure as predictors of future psychological outcomes, we may consider them as instigators of a chain of processes that influence social relationships and consequent health and well-being throughout the life course.

## Relational (and Contextual) Nature of Identities and Authenticity

According to the developmental model of the closet, one might conclude that some identities are more “real” than others. For example, Pachankis and Jackson (2022) speak of becoming “truly known by others” through disclosure, implying that the identity one projects before disclosure is not as authentic or real as the one presented after. While we acknowledge that some individuals might experience their identity in this way, this is unlikely to be a universal experience. Crucially, we argue that identities are responsive to relational changes in environments (Doyle, 2022), which has implications for what it means to be authentic.

The idea that being authentic requires coming out assumes there is only one fixed way to see oneself that qualifies as authentic. Instead, identities encompass a flexible and malleable portfolio of self-aspects, only some of which are emphasized in any given context. As set out in detail by self-categorization theory (Turner et al., 1987), each individual has multiple identities and not all of them are relevant or important in each context. One’s sexual minority identity might be salient and relevant when discussing inclusivity issues, but it is probably one’s identity as a lecturer that is likely to be at the forefront when one is teaching a class. Such contextual cues make different aspects of the self more or less salient and relevant, even though both salient and non-salient self-aspects might be equally authentic representations of the self. Furthermore, sexual identities might genuinely vary across contexts and relationships (Katz-Wise & Todd, 2022). For example, rather than always reflecting a latent sexual minority identity that has been concealed or denied, people may find themselves questioning their sexual orientation because of very specific attractions to, or relationships with, specific individuals (Brown, 2009; Katz-Wise & Todd, 2022; Lewis et al., 2022); so the fact that a particular identity is not being overtly expressed in a specific context does not mean it is not real.

In addition, stating that concealment impairs authenticity assumes that sexual orientation is the most important aspect of one’s self-view. However, as Pachankis and Jackson (2022) acknowledge, research has shown variability in the extent to which individuals find their sexual minority identity central and, furthermore, demonstrated that this variability in identity centrality determines the extent to which disclosure is linked to feelings of authenticity (Fletcher & Everly, 2021). Specifically, when an individual holds multiple identities that are perceived as incompatible, concealing a stigmatized identity can constitute a deliberate strategy to ensure an alternative representation of the self that one considers to be equally authentic and contextually or relationally important. For example, gay Jewish men sometimes find that expressing their Jewish identity requires concealing their sexual orientation (Faulkner & Hecht, 2011). Similarly, queer people of color who perceive their sexual orientation and ethnicity to be incompatible sometimes decide to conceal from family (Sarno et al., 2015), and while they might be doing so to avoid sanction, they might also be doing so because that allows them to be regarded by others simply as a family member. In this way, concealment can be liberating, freeing individuals to express valued identities unimpeded by stereotypes associated with other (potentially stigmatized) aspects of one’s identity, such as sexual orientation (Barreto et al., 2006; Newheiser & Barreto, 2014). More broadly, intersectional identities complicate simple master narratives of “coming out” (e.g., Ghabrial, 2017).

It is important to stress that although ideally there would be no stereotypes or prejudice with which to contend, the option currently available to those with stigmatized identities is not to disclose all identities without peril, but to manage their self-presentation in ways that allow each identity to take its place. More broadly, these ideas point to the need to move beyond normative notions that disclosure is always ideal, which feed into what has been designated as “concealment stigma” (Le Forestier et al., 2022), whereby strong expectations of disclosure across contexts lead to a moralization of concealment that punishes members of stigmatized groups who do choose to conceal for whatever reason (Doyle, 2022). This is not to say that individuals are completely in control of their own identities, which they manage in ways that secure authenticity. Indeed, we acknowledge that people often report that concealment impairs authenticity (Newheiser & Barreto, 2014). Instead, what we argue is that managing stigmatized identities often involves a degree of controlled self-expression that takes into account external views of the self (Barreto & Ellemers, 2003; Deaux & Major, 1987; Doyle, 2022; Swann, 1987). Moreover, these external views define both category boundaries and their contents, influencing not only self-expression, but even what an individual regards as a possible identity, and what that means to them. Indeed, societal views about sexual minorities can influence even whether one identifies as gay, not just due to fear of sanction, but also due to one’s inability to imagine a self that matches the stereotype (King & Smith, 2004).

We also do not mean to convey that identity expression is unimportant. By expressing one’s identity, it becomes real to specific others, who then become able to respond to it in particular ways, sometimes challenging how we see that identity (e.g., Garr-Schultz & Gardner, 2021), and at other times validating it (e.g., Doyle et al., 2021). Identities that remain unexpressed cannot be explored, rehearsed, challenged, in sum, shaped in the same way as those that are enacted (Hopkins & Greenwood, 2013). But crucially, one cannot say that this identity exploration or challenge only happens the first time one discloses one’s identity, as suggested by the developmental model of the closet. Each disclosure affects identities in unique ways, both because different people respond in different ways to the same disclosure and because not everyone’s reactions matter in the same way. This iterative process of disclosure and self-change also means that disclosure is different every time in part because the self will have changed through prior experiences.

The notion that identities are shaped through social interaction also leads us to question the idea that there is a fixed order of events in which one self-labels first and comes out after (cf. Pachankis & Jackson, 2022). Since identities develop in part through disclosure and self-expression, self-labeling can happen after, or go hand in hand with, coming out. In some cases, identities hardly even exist prior to social interaction. Collective identities (and their labels), for example, often emerge through expression, as people find themselves in the same place, doing similar things, with little prior self-categorization or idea of what the identity was about, as has been well investigated in the psychology of crowds (Drury & Reicher, 2000). At times, one might even self-label because others have pointed it out, in a kind of reversed disclosure. Crowd members often become identified with the crowd by onlookers, who then treat them as a homogeneous entity, leading to an initial self-categorization (Reicher, 1984, 2004). Similarly, young people might not be aware of the various ways in which sexual orientation plays out before they start being identified by others as a sexual minority. Indeed, Pachankis and Jackson (2022) are correct to note that parents, siblings, peers and others often notice a “difference” before one outwardly, or sometimes inwardly (i.e., to oneself), identifies as a sexual minority (e.g., D’Augelli et al., 2008). Importantly, if self-change often results from identity expression and disclosure, and if identities often develop through expression, then it is hard to specify what identity is authentic—the one we had in our mind prior to expressing it, or the one we have after interacting with others. Precisely because of all these difficulties determining what the true self is, some have argued that authenticity should not necessarily be regarded as being true to oneself, but as one’s subjective feeling that one is being true to oneself (Rivera et al., 2019).

It is also important to consider that different cultural notions of selfhood, and relational selves (Markus & Kitayama, 1991), can play a role in what people consider to be their true or authentic self. Cultural norms influence the identities people value, as well as the extent to which they are comfortable with contradictory beliefs about themselves (Choi & Choi, 2002). For example, some East Asian cultures place great value on adjusting to the social environment, causing fluctuations in self-concept and behavior, whereas people in many Western cultures find such variation indicative of a lack of authenticity (English & Chen, 2007). As such, it is possible that sexual identity disclosure is more closely related to feelings of authenticity in Western than Eastern societies. If so, then one might also advance that coming out might have very different consequences to how one views oneself depending on the cultural context (e.g., Huang & Brouwer, 2018).

## Conclusion

We argue for the need to more thoroughly consider relational dynamics both in the conceptualization of sexual minority identity concealment and in the understanding of sexual minority identities. Coming out is best seen as a process, rather than a moment, which can be impactful, but also rather unremarkable, depending on others’ responses. Identities are best seen as multiple, flexible, and responsive to the views of others in one’s social environment, with authenticity meaning different things to different people, rather than being predicated on disclosure. Perhaps it is time to retire the metaphor of the static closet for sexual minorities; a more apt metaphor may be a trunk that is moveable from one location to another and from which one emerges again and again, each time potentially bringing in and out new items as one goes.
